# Chimeric RNA in Cancer and Stem Cell Differentiation

**DOI:** 10.1155/2018/3178789

**Published:** 2018-10-28

**Authors:** Justin Elfman, Hui Li

**Affiliations:** ^1^Department of Biochemistry and Molecular Genetics, University of Virginia, 22903, USA; ^2^Department of Pathology, University of Virginia, 22903, USA

## Abstract

Gene fusions are considered hallmarks of cancer which can be produced by chromosomal rearrangements. These DNA-level fusion events may result in the expression of chimeric RNAs; however, chimeric RNAs can be also produced by intergenic splicing events. Chimeric transcripts created by the latter mechanism are regulated at the transcriptional level and thus present additional modes of action and regulation. They have demonstrated importance in normal cell physiology, and their dysregulation can induce oncogenesis and impact cell differentiation. In this review, we outline proven mechanisms through which intergenically spliced chimeric RNAs are involved in carcinogenesis. We highlight their similarity to canonical chimeric RNAs resulting from gene fusions as well as their unique qualities. Additionally, we review known roles of chimeric RNA in cell differentiation and propose means through which chimeric RNAs may be valuable as stage-specific markers or as targets for expression profiling.

## 1. Introduction

Chimeric RNAs are transcripts comprising the nucleotide sequence from different parental genes [1–5]. These transcripts are known to not only be produced by gene fusion but can also be formed via intergenic splicing events. Intergenically spliced chimeric RNAs have been shown to occur via *cis*-splicing of adjacent genes (*cis*-SAGe) as well as long-range intrachromosomal and interchromosomal *trans*-splicing events [3, 5, 6]. While specific mechanisms for intergenically spliced chimeric RNA generation are unclear, some recurring patterns have emerged. For instance, in *cis*-SAGe chimeras, most transcripts follow the 2-2 rule, where the penultimate exon of the 5′ gene is spliced to the second exon of the 3′ gene [7, 8], and several occurrences of intergenic *trans*-splicing have been found to occur between neighboring genes on opposite strands [9–12]. Both patterns suggest potential importance of parental gene proximity in chimeric RNA production. Despite their mysterious origin, intergenically spliced chimeric RNAs are found across tissue types and have proven importance in normal cell states [2, 3, 5, 8] as well as demonstrated roles in both oncogenesis and cell differentiation. While similar in concept to chimeric transcripts created by gene fusion, transcription-level processing presents additional functionality and nuanced regulation unique to intergenically spliced chimeras. In this review, we present several examples of similarities between both sources of chimeric RNA as well as these differences. We also present examples of chimeric RNAs involved in oncogenesis and cell differentiation as well as further possible mechanisms for the role of chimeric RNA in these events. Finally, we highlight the potential of chimeric RNA to serve as a cell type and stage-specific marker for expression profiling.

## 2. Gene Fusion and Fusion Transcripts

In this manuscript, we refer to chimeric RNAs generated by gene fusions as *fusion transcripts*. These transcripts are typically transcribed from abnormal genomic regions created by chromosomal rearrangement rather than by intergenic splicing. Gene fusions are often distinctive features of particular cancer types and generate cytogenetic signatures characteristic of different malignancies. These have been successfully used as diagnostic markers [13, 14] as well as therapeutic targets [15–17].

This is perhaps best exemplified by the *BCR-ABL1* fusion, which encodes a novel tyrosine kinase in chronic myelogenous leukemia [16]. The BCR-ABL1 fusion protein provides additional regulatory binding domains contained within BCR to the ABL1 tyrosine kinase, which increases the number of potential targets for the kinase [16]. The *BCR-ABL1* fusion has been used as a biomarker as well as a therapeutic target by the drug imatinib, which binds specifically to the kinase active site. As a result, patients diagnosed within the *BCR-ABL1* subtype have favorable prognoses [15, 16, 18].

Gene fusions can also induce oncogenesis without producing a novel protein. One such example combines the 5′ UTR of *TMPRSS2* to a member of the *ETS* transcription factor family (*TMPRSS2-ETS*). TMPRSS2 is a serine protease which is upregulated in response to androgen activation. The ETS family of transcription factors regulates a multitude of key cellular processes, and dysregulation can result in oncogenesis. ETS is overexpressed in 50% of all prostate cancers, of which 90% exhibit the *TMPRSS2-ETS* fusion. This fusion introduces an androgen-responsive regulatory element to ETS, which upregulates the ETS expression in response to androgen activation, leading to oncogenesis [14, 19, 20].

## 3. Intergenically Spliced Chimeric RNAs

**Figure 1:**
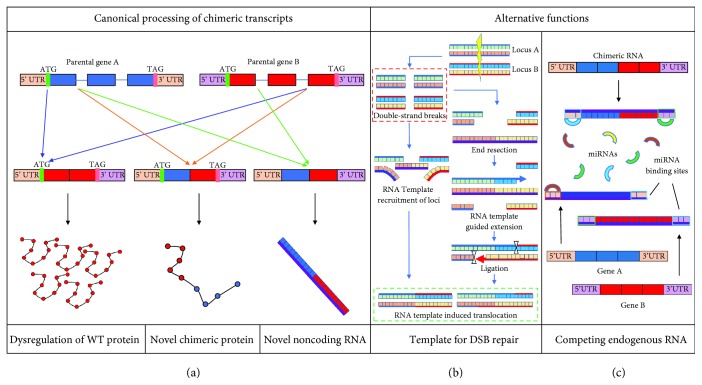
Implications of chimeric RNA in oncogenesis. (a) Canonical processing of chimeric transcripts. Colored rectangles represent exons, and connecting lines represent introns. Colored arrows indicate splicing configuration. Circles represent amino acids, and the nucleic acid with a purple backbone represents a mature mRNA transcript. Canonical processing includes dysregulation of a wild-type protein via splicing an ectopic UTR to a wild-type coding sequence, splicing of two in-frame coding sequences to produce a novel protein, and splicing into long noncoding RNA. (b) Chimeric RNA as a template for DSB repair. Two possible mechanisms are presented: chimeric RNA can serve as a template to recruit two distant genomic loci into proximity; chimeric RNA can serve as a homologous template for translocation of two distant genomic loci. (c) Chimeric RNA as ceRNA. Chimeric transcripts retain sequence homology with parental genes, thus potentially retaining miRNA binding sites to compete for local miRNAs.

Similar to fusion transcripts, chimeric RNAs generated by intergenic splicing can give rise to fusion proteins, which reflect the combined coding sequence of its parental genes (Figure 1(a)). Some of these transcripts are identical to those created by hallmark gene fusion events, which produce oncogenic proteins. Events which create these gene fusions at the DNA level result in constitutive overexpression of the chimeric RNA and therefore overexpression of the novel fusion protein. One prominent example is the *JAZF1-JJAZ1* gene fusion prevalent in endometrial stromal sarcoma. Both the chimeric RNA and protein are also present in normal endometrial stromal cells, and overexpression of the protein confers antiapoptotic activity, promoting cell survival [1, 21].

Intergenically spliced chimeric RNAs have also been shown to utilize the ETS family of transcription factors. Several such examples have been published including the *SLC45A3-ELK4* chimeras. Similar to *TMPRSS2*, *SLC45A3* (solute carrier family 45, member 3) is an androgen-responsive gene specifically expressed in the prostate. Rickman et al. described a chimera joining exon 1 of *SLC45A3* to exon 2 of *ELK4*. Notably, as *SLC45A3* exon 1 does not contain a coding sequence, the chimeric RNA adopts an androgen-responsive 5′ untranslated region while coding for wild-type ELK4 [22]. Maher et al. detected an isoform which joined *SLC45A3* exon 4 to *ELK4* exon 2 and also showed association with prostate cancer [23]. Further characterization of *SLC45A3-ELK4* chimeras showed that the transcript was created through *cis*-SAGe rather than *trans*-splicing [24], and most notably the exon 1/exon 2 form of the chimera functions as an androgen-responsive chimeric long noncoding RNA [25] (Figure 1(a)).

## 4. Chimeric RNA as Potential Templates for RNA-Guided DSB Repair and Rearrangement

An overlap between common loci for chromosomal translocation and parental genes involved in intergenic splicing may not be coincidental. As chimeric *trans*-splicing requires both parental transcripts to be present, it is likely that these events may be dependent upon the spatial proximity of the parental genes. It is well known that three-dimensional proximity of genomic regions increases the likelihood for translocation to occur between those regions through erroneous repair following double-strand breaks (DSB) [26–28]. Specific examples include *BCR-ABL1* and *MYC-IGH*, which are hallmarks of chronic myelogenous leukemia and Burkitt's lymphoma, respectively [28]. RNA templates or corresponding cDNA have been shown to mediate homologous recombination and DSB repair in the absence of a homologous chromosome [29–31]. Several authors have suggested that *trans*-spliced chimeric RNA or reverse-transcribed chimeric cDNA may serve as template for DNA rearrangement [3, 9, 32, 33], which would provide another mechanism for the induction of DNA-level gene fusion (Figure 1(b)). The occurrence of chimeric transcripts such as *JAZF1-JJAZ1* and *PAX3-FOXO1* (described hereafter), in both normal and neoplastic cells, supports this possibility.

## 5. Chimeric RNA as Potential Competing Endogenous RNA

In addition, similarity in sequence to parental genes presents chimeric RNAs as candidates to serve as competing endogenous RNAs (ceRNA), or micro RNA (miRNA) sponges, for both parental genes (Figure 1(c)). Recently, competing functions of transcribed noncoding regions of the genome have been described which are affected in certain subtypes of cancer [34]. Particular emphasis is placed on transcribed pseudogenes due to sequence homology, tissue-specific expression, and evolutionary conservation despite their lack of coding functionality [34–36]. Typically, ceRNAs are thought to compete with other transcripts of similar sequence by means of common miRNA binding sites. miRNA regulation has been implicated in many cancers, among other diseases [37, 38], and dysregulation of ceRNAs such as *HULC* or *PTENP1* can lead to oncogenesis [35, 39].

## 6. Chimeric and *Trans*-Spliced RNAs in Stem Cell Differentiation

Stem cell differentiation is generally considered a sequential process in which cells acquire new characteristics. These changes largely occur without alteration to the genome. Instead, iterative changes to the epigenome, primarily driven by the action of transcription factors (TF), coordinate cell fates [40–43]. Chromatin accessibility changes through the course of cell differentiation, induced by TF specific to cell type. These TF have been used to generate profiles indicative of cell stages through differentiation [40, 43]. TF regulation can affect cell differentiation [42, 44, 45] and can produce undifferentiated or dedifferentiated phenotypes characteristic of certain cancers [42, 45–47]. Further, the genome undergoes significant changes in higher-order chromatin organization through stages of differentiation [48, 49], which affect interaction frequencies between gene compartments as well as genes within these compartments.

Any of these changes have the potential to disrupt or introduce expression of chimeric RNAs. Thus, many chimeric transcripts show considerable tissue specificity [5, 50], several of which have been shown to be upregulated in cancer [4, 22–24, 51]. A subset of these are regulated through cell differentiation and can consist of TF parental genes [50]. One such example is a chimeric *PAX3-FOXO1* transcript which is formed through joining the DNA-binding domain of PAX3 to the transactivation domain of FOXO1 [52]. This chimeric RNA is identical in form to the *PAX3-FOXO1* hallmark gene fusion found in alveolar rhabdomyosarcoma (ARMS), a small blue round cell tumor with characteristic undifferentiation. The transcript is translated into a novel TF which regulates genes involved in myogenesis, myogenic signaling, and mesodermal development [53], and it has been shown to interfere with normal PAX3 and FOXO1 activity [54]. Chimeric *PAX3-FOXO1* is regulated through myogenesis, and its dysregulation interferes with proper differentiation [55].

Alternative intragenically *trans*-spliced RNAs (tsRNA) have also been shown to regulate embryonic stem cell differentiation. Through applying stringent criteria to predicted chimeric products, Wu et al. uncovered four noncollinear *trans*-spliced mRNAs which exhibited differential expression between *trans*-spliced and wild-type isoforms as well as differentiated and undifferentiated cell types. The tsRNAs also showed differing tissue specificity when compared to the wild-type transcripts and showed that the knockdown of one such long noncoding tsRNA impaired pluripotency maintenance through interaction with pluripotency-associated factors NANOG and SUZ12 [56].

## 7. Chimeric RNA Expression Profiling

**Figure 2:**
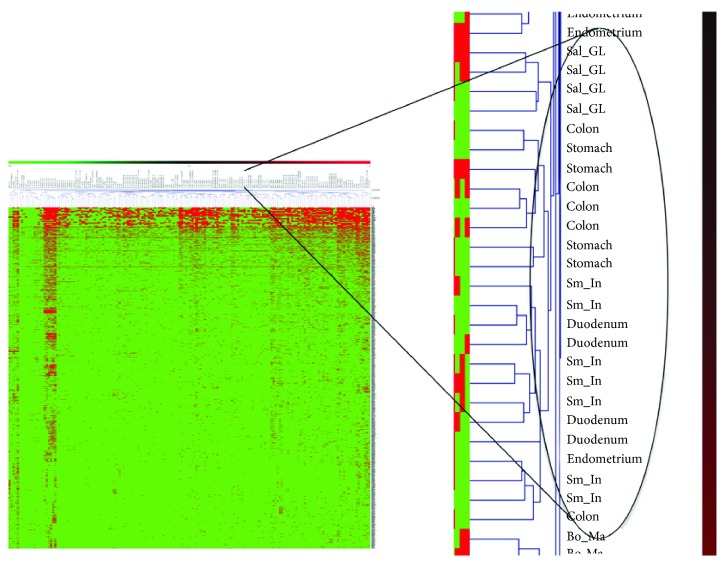
Unsupervised clustering of 27 nonneoplastic tissue samples from 171 RNA-Seq libraries by chimeric RNA expression group tissues with common developmental origin.

Tissue and cell-stage specificity of chimeric RNAs and tsRNAs provides a strong basis for expression profiling. In fact, chimeric RNA profiling has been successfully used as a means to cluster cells from similar nonneoplastic tissue types [50] (Figure 2). Further, chimeric RNAs offer a unique opportunity to identify unknown cell of origin in undifferentiated tumor types. Exploring this possibility in ARMS, Xie et al. performed chimeric transcriptome profiling at four time points throughout myogenesis and found that the majority of chimeric RNAs were generated transiently and exclusively during differentiation. They were able to determine the chimeric RNA profile of RH30, an ARMS cell line, and found a set of 18 chimeric RNAs which appeared to be uniquely expressed by RH30 at one specific time point during myogenesis [50]. These findings are also in agreement with time-specific expression of myogenic expression of *PAX3-FOXO1* in an earlier study [2]. This methodology offers another valuable perspective to identifying cell of origin and may provide insight into other mysterious tumors.

## 8. Conclusion

Several exemplary cases of chimeric RNAs presented herein are shown to play roles in crucial cell processes; these are not likely isolated phenomena. Recurrent chimeric RNAs have been predicted and validated across various tissue types, and several have shown functional relevance in cell proliferation or motility [5, 24, 51]. The presence of chimeric RNAs in cancer and precancer lesions supports their potential as biomarkers and therapeutic targets. Chimeric transcripts may provide means for oncogenesis in cancers with notably low mutational burden such as acute myeloid leukemia [57, 58] or are perhaps veiled contributing factors to cancers with multiple oncogenic sources. Further, if proposed mechanisms for chimeric RNA-templated chromosomal translocation and activity as ceRNA are found to be true, controlled regulation of chimeric transcripts could play an important role in preventative cancer treatment.

Tissue and cell-stage specificity provides additional utility for the use of chimeric RNAs as diagnostic indicators. Increased accuracy in tissue profiling studies could improve specificity in treatments targeting particular cell types, and chimeric RNAs offer another avenue towards this end. The presence of chimeric RNAs which mirror hallmark cancerous fusions in precancer cells could also provide information on the cell of origin for mysterious tumors. Further, cell-stage specificity of chimeric RNA expression may give insight into particular pathologies for cancer progression.

In summary, while the mechanisms for chimeric RNA creation are not entirely clear, their importance and dynamic functionality continue to be proven. Chimeric RNA presents an underexplored library of biomarkers and regulatory pathways which could improve clinical treatment, provide insight into unknown oncogenic pathologies, and help to understand or classify mysterious differentiation states and tissues of origin.
